# Case of Bi-Partite Vagina; with Spontaneous Rupture of the Septum, and Natural Delivery

**Published:** 1845-01

**Authors:** 


*Case of Bi-Partite Vagina; with Spontaneous Rupture of the Septum, and Natural Delivery.* By Dr. Vinchon.—In August, 1842, I was called by a midwife to examine the genital organs of a primipara who had been in labour, from one evening to the next. The head, which was in the first position, was far down in the pelvis; and on a cursory examination, every thing gave promise of a speedy and favourable issue; but in carefully examining the vagina, a thick septum was detected, stretching vertically between the recto-vaginal partition, and the urethro-vaginal wall, beginning on a level with the posterior commissure of the urinary meatus, and terminating about two inches beneath it. The vagina was thus divided into a right and left canal. The mouth of the womb was quite free, because at the end of the septum, the vagina was single. The anormal condition just described gave rise to no inconvenience in sexual intercourse, as the penis could be introduced indiscriminately into one or other canal. The finger could be put in at one opening, carried round the septum, and brought out at the other.

As labour was advancing slowly, and the septum could at any time be prevented from impeding delivery by snipping it across with scissors, I satisfied myself by administering ergot of rye, and watching to see what nature would do. The head was neither directed to one side or the other by the septum, which by the force of the uterine contractions, was made to appear like a band across the head, in thickness similar to the umbilical cord. As the expulsive force increased, the band became thinner: from the absence of blood, and the effects of tension, it was rendered white like a ligament. Our intention now was to destroy this obstacle by cutting it through; but nature was more alert. All at once the membrane snapped across towards the anterior commissure of the vulva, yielding a noise resembling the slight crack of a whip, whereupon the head immediately came forth, and a healthy child was soon born. The portion of membrane attached to the posterior commissure was cut off with scissors.—*Lond. and Ed. Monthly Journ., from L'Expérience.*

